# Safety and Feasibility of Fasting-Mimicking Diet and Effects on Nutritional Status and Circulating Metabolic and Inflammatory Factors in Cancer Patients Undergoing Active Treatment

**DOI:** 10.3390/cancers13164013

**Published:** 2021-08-09

**Authors:** Francesca Valdemarin, Irene Caffa, Angelica Persia, Anna Laura Cremonini, Lorenzo Ferrando, Luca Tagliafico, Alberto Tagliafico, Ana Guijarro, Federico Carbone, Stefano Ministrini, Maria Bertolotto, Pamela Becherini, Tommaso Bonfiglio, Chiara Giannotti, Amr Khalifa, Moustafa Ghanem, Michele Cea, Marzia Sucameli, Roberto Murialdo, Valentina Barbero, Raffaella Gradaschi, Francesca Bruzzone, Consuelo Borgarelli, Matteo Lambertini, Claudio Vernieri, Gabriele Zoppoli, Valter D. Longo, Fabrizio Montecucco, Samir G. Sukkar, Alessio Nencioni

**Affiliations:** 1 Department of Internal Medicine and Medical Specialties, University of Genoa, 16132 Genoa, Italy; francesca.valdemarin@gmail.com (F.V.); irene.caffa@libero.it (I.C.); angelica.persia@gmail.com (A.P.); annalauracremonini@gmail.com (A.L.C.); lor.ferrando@gmail.com (L.F.); tagliaficoluca1992@gmail.com (L.T.); federico.carbone@unige.it (F.C.); maria.bertolotto@unige.it (M.B.); bonfigliot@gmail.com (T.B.); chiara.giannotti@hsanmartino.it (C.G.); amr.khalifa@edu.unige.it (A.K.); moustafa.ghanem@edu.unige.it (M.G.); michele.cea@unige.it (M.C.); sucamelimarzia@virgilio.it (M.S.); consuelo.borgarelli@unige.it (C.B.); matteo.lambertini@unige.it (M.L.); gabriele.zoppoli@unige.it (G.Z.); fabrizio.montecucco@unige.it (F.M.); 2 IRCCS Ospedale Policlinico San Martino, 16132 Genoa, Italy; albertotagliafico@gmail.com (A.T.); anaguijarroa@yahoo.es (A.G.); pamy.bekke@gmail.com (P.B.); rozzis752000@yahoo.it (R.M.); valentina.barbero@hsanmartino.it (V.B.); raffaella.gradaschi@hsanmartino.it (R.G.); francesca2.bruzzone@hsanmartino.it (F.B.); samir.sukkar@hsanmartino.it (S.G.S.); 3 Center for Molecular Cardiology, Universität Zürich, 8952 Schlieren, Switzerland; stefano.ministrini@uzh.ch; 4 Internal Medicine, Angiology and Atherosclerosis, Department of Medicine and Surgery, Università degli Studi di Perugia, 06129 Perugia, Italy; 5 IFOM, FIRC Institute of Molecular Oncology, 20139 Milan, Italy; claudio.vernieri@ifom.eu (C.V.); vlongo@usc.edu (V.D.L.); 6 Medical Oncology and Hematology Department, Fondazione IRCCS Istituto Nazionale dei Tumori, 20133 Milan, Italy; 7 Longevity Institute and Davis School of Gerontology, University of Southern California, Los Angeles, CA 90089, USA

**Keywords:** modified fasting, cancer metabolism, feasibility, safety, circulating growth factors, body composition

## Abstract

**Simple Summary:**

Our phase I/II clinical trial demonstrated that periodic cycles of a modified fasting regime (“fasting-mimicking diet” (FMD) by L-Nutra) were feasible and safe in cancer patients at low nutritional risk and that they did not negatively affect the patients’ body composition when combined with dietary and muscle training instructions to promote lean body mass re-gain in the periods between FMD cycles. While previously published studies of modified fasting in cancer patients exclusively enrolled patients receiving chemotherapy, in our trial, we also accrued patients treated with different types of therapies, including endocrine therapies (w/ or w/o CDK4/6 inhibitors), TKIs, proteasome inhibitors, immune check point inhibitors and radiotherapy. Eighteen different types of cancer are represented in our trial. Our study also shows that the FMD decreased fat mass and effectively lowered the circulating insulin, IGF1 and leptin. These are important elements of novelty of our study when compared to previously published trials.

**Abstract:**

In preclinical studies, fasting was found to potentiate the effects of several anticancer treatments, and early clinical studies indicated that patients may benefit from regimes of modified fasting. However, concerns remain over possible negative impact on the patients’ nutritional status. We assessed the feasibility and safety of a 5-day “Fasting-Mimicking Diet” (FMD) as well as its effects on body composition and circulating growth factors, adipokines and cyto/chemokines in cancer patients. In this single-arm, phase I/II clinical trial, patients with solid or hematologic malignancy, low nutritional risk and undergoing active medical treatment received periodic FMD cycles. The body weight, handgrip strength and body composition were monitored throughout the study. Growth factors, adipokines and cyto/chemokines were assessed by ELISA. Ninety patients were enrolled, and FMD was administered every three weeks/once a month with an average of 6.3 FMD cycles/patient. FMD was largely safe with only mild side effects. The patients’ weight and handgrip remained stable, the phase angle and fat-free mass increased, while the fat mass decreased. FMD reduced the serum c-peptide, IGF1, IGFBP3 and leptin levels, while increasing IGFBP1, and these modifications persisted for weeks beyond the FMD period. Thus, periodic FMD cycles are feasible and can be safely combined with standard antineoplastic treatments in cancer patients at low nutritional risk. The FMD resulted in reduced fat mass, insulin production and circulating IGF1 and leptin. This trial was registered on Clinicaltrials.gov in July 2018 with the identifier NCT03595540.

## 1. Introduction

Mouse studies show that periodic cycles of fasting enhance the activity of several types of anticancer agents, such as cytotoxic chemotherapy, endocrine agents used in breast cancer treatment and tyrosine kinase and immune checkpoint inhibitors [1,2,3,4]. Both cell-autonomous antitumor effects, such as the impaired Warburg effect, predisposition to experience oxidative stress, MAP kinase and AKT-mTOR signaling inhibition or enhanced p53 activity, and indirect effects, such as enhanced tumor cell immunogenicity, have been implicated in the fasting-induced sensitization of neoplastic cells to anticancer treatments [4,5,6,7,8]. 

In addition to its antitumor effects, fasting was shown to protect healthy tissues in mice from chemotherapy-mediated damage, including cardiotoxicity, via the induction of a self-protection mode, which, in turn, reflects the upregulation of resistance-enhancing transcription factors, such as early growth response 1 (EGR1) [9,10]. Fasting-induced enhancement of chemotherapy efficacy in tumor models was initially proposed to be mediated in part by reduced blood insulin growth factor 1 (IGF1) and glucose levels [7]. However, in a recent study, we demonstrated that the fasting-mediated enhancement of endocrine therapy activity in hormone receptor-positive breast cancer (HR+ breast cancer) also relies on the ability of fasting to blunt circulating insulin and leptin concentrations [2]. On the other hand, the IGF1- and sugar-lowering effects of fasting were shown to be key for its ability to reduce the toxicity of chemotherapeutics [9,11].

On these bases, several clinical studies have explored the feasibility, safety and potential benefits of fasting in patients receiving concomitant anticancer treatments. These clinical trials have either utilized water-only fasting for periods ranging between one and seven days, or modified fasting regimes [2,12,13,14,15,16,17]. The latter have been either in the form of a low-calorie, low-sugar, low-protein diet (typically consisting of vegetables, vegetable juices/soups, nuts, herbal teas, etc.) prescribed to patients [12,17] or of kits containing the food and the vitamins that the patient is supposed to eat to recreate the metabolic effects of fasting (a “Fasting-Mimicking Diet”—FMD) [2,13,18,19]. 

These studies have shown that both water-only fasting and modified fasting regimes are generally feasible and safe, with essentially no cases of severe adverse events (i.e., G3–5 according to the Common Terminology Criteria for Adverse Events (CTCAE)) due to these dietary interventions. In three of these studies, enrolling patients with breast cancer or gynecological malignancies, modified fasting was associated with better quality of life/patient reported outcomes during chemotherapy [12,17,20]. In the study by Zorn and colleagues, a modified fasting regime was found to alleviate chemotherapy-emergent stomatitis as compared to an *ad libitum* diet [17]. 

However, benefits of modified fasting in terms of reduced G3/4 global adverse events, neutropenia and neutropenic fever were not found in the DIRECT randomized clinical trial, which enrolled breast cancer patients undergoing neo-adjuvant chemotherapy w/ or w/o FMD cycles [13,20]. It is noteworthy that in the study by De Groot and colleagues, a radiological complete or partial response and pathological responses occurred more often in patients who were compliant with the FMD [13]. We recently reported a partial analysis of the NCT03595540 clinical study of a FMD regime in cancer patients performed at our hospital in Genoa (Italy) [2]. 

This trial enrolled patients at low nutritional risk and undergoing active anticancer treatment. In our recent article, we presented data from patients with breast cancer receiving endocrine therapy and FMD cycles (patients #1–#24) and showed that the FMD effectively lowered the serum levels of growth-promoting and pro-oncogenic factors, such as insulin, IGF1 and leptin, with IGF1 and leptin remaining low for weeks beyond the FMD period [2]. Thus, overall, albeit preliminary, the available data on modified fasting regimes in cancer patients undergoing standard treatments point towards potential advantages of this dietary approach, which require confirmation in larger clinical trials.

Concerns have been raised that fasting/modified fasting may pose substantial risks for cancer patients in terms of detrimental effects on their nutritional profile, potentially leading to malnutrition and an impaired immune system in predisposed subjects [21,22]. When monitoring body composition in gynecological cancer patients undergoing cycles of modified fasting, Zorn and coworkers did notice a progressive reduction in the mean body cell mass and in the mean phase angle [17]. Thus, these findings indicate that the type of modified fasting regime applied in this clinical trial indeed induced unfavorable changes in body composition. 

In our study in breast cancer patients receiving endocrine agents, when combined with dietary and muscle training instructions to promote weight and lean body mass re-gain in the periods between one FMD cycle and the next one, the FMD was associated with favorable changes in bioimpedance phase angle and in body composition [2]. Namely, this multimodal approach that we adopted was found to eventually increase phase angle value and lean body mass, while decreasing fat mass. However, whether similar results are also obtained with the FMD in patients undergoing chemotherapy or other treatments, which are at higher risk to cause anorexia and malnutrition remains to be defined.

Based on these premises, the aim of our study was to evaluate the overall feasibility, safety and impact on body composition of a FMD in the NCT03595540 clinical study, as well as the effect of this FMD on circulating growth-promoting factors, adipokines and on cyto/chemokines with a role in cancer and inflammation.

## 2. Materials and Methods

### 2.1. Patients

In this single-arm, phase I/II clinical trial (NCT03595540), eligible patients of both sexes had a diagnosis of solid or hematological tumors, adequate nutritional status (i.e., body mass index (BMI) > 19.0 kg/m^2^, low (0–1) nutritional risk screening (NRS) scores, bioimpedance phase angle > 5°), normal organ function (liver, heart, kidney), ECOG performance status of 0 or 1) and age >18 years, and were in active treatment for their tumors. Patients were excluded if they had a diagnosis of diabetes, allergies for components of the diet or if they received other experimental treatments. 

The study was conducted in accordance with the Declaration of Helsinki (October 2013) at the IRCCS Ospedale Policlinico San Martino (Genoa, Italy), after protocol approval by the Ethics Committee of the Regione Liguria. An amendment to the protocol, which included the possibility to enroll 90 patients, rather than the initially planned 60 patients, and to use imaging studies that are otherwise routinely prescribed for disease monitoring (e.g., CT scans for body composition evaluation), was approved by the same ethics committee on 8 April 2019. Patients were enrolled from November 2017 to August 2020. All patients provided written informed consent before inclusion.

### 2.2. Anticancer Treatments

Anticancer treatments allowed for the study were chemotherapy (i.e., doxorubicin, paclitaxel, carboplatin, cisplatin, capecitabine, temozolomide, cyclophosphamide, vinorelbine, eribulin, gemcitabine, taxol, mitomycin C, etoposide, oncocarbide, XELOX, FOLFOX and EC), radiotherapy, endocrine therapy (steroidal or non-steroidal aromatase inhibitors (i.e., letrozole, exemestane and anastrozole), tamoxifen, fulvestrant and GnRH analogues)), other molecular targeted therapies (including tyrosine kinase inhibitors (TKIs, i.e., ruxolitinib, nintedanib and nilotinib), CDK4/6 inhibitors (i.e., abemaciclib, ribociclib and palbociclib), proteasome inhibitors (i.e., carfilzomib), immunomodulatory drugs (i.e., lenalidomide), biological drugs (i.e., trastuzumab, T-DM1, pertuzumab and bevacizumab) and immune checkpoints inhibitors (i.e., pembrolizumab, ipilimumab and durvalumab)), glucocorticoids (dexamethasone) and Bacillus Calmette-Guerin (see Supplementary Table S1).

### 2.3. Intervention

The FMD (supplied by L-Nutra Inc., Los Angeles, CA, USA) is a 5-day, low-calorie and low-protein diet that supplies approximately 4600 kj (1099 kcal) on Day 1 (11% protein, 46% fat and 43% carbohydrates), approximately 3000 kj (717 kcal) (9% protein, 44% fat and 47% carbohydrates) on Days 2–5 and which consists of plant-based ingredients all generally recognized as safe (GRAS) according to the FDA. In chemotherapy-treated patients the diet was typically administered 4 days prior to and on the day of each chemotherapy cycle, while, in other patients, the FMD was administered once a month, regardless of concomitant anticancer treatments. 

Patients underwent FMD cycles of lower duration (3- or 4-day FMD) if phase angle decreased to values comprised between 5.2° and 5.0°. If the phase angle was <5°, the corresponding FMD cycle was not administered, and the patient was re-evaluated after 4 weeks and eventually supplemented with essential amino acids (Aminotrofic^®^: 5.5 g b.i.d.). 

In the time interval between subsequent FMD cycles, patients were encouraged to follow a personalized recovery diet regime with adequate caloric (20–30 kcal/kg weight/day) and protein intake (1.2–1.5 g protein/kg weight/day, mainly derived from fish, legumes, eggs and dairy products), and they were also invited to carry out a mild-to-moderate daily muscle training (20–30 min, 500–600 kj/day) according to the instructions provided to the patients (https://docs.google.com/presentation/d/1szaRW4t-pZQI17o2Pe0747FsUEOVWWiA6BHja0p8H_0/edit?usp=sharing accessed on 5 August 2021).

The latter was modulated based on patient clinical conditions, with the aim of promoting protein anabolism and the preservation/recovery of muscle mass. During the first six months on treatment, FMD cycles were prescribed monthly or every three weeks at the most. Thereafter, the patients were allowed to shift to a bimonthly schedule or to receive the FMD in combination with every other cycle of chemotherapy given q21 days.

### 2.4. Feasibility and Safety Assessment

The primary study objective was to assess the feasibility and safety of the FMD. The feasibility of FMD, which was monitored through telephone interviews or the analysis of patients’ food diaries, was defined as strict adherence to the prescribed diet with the possibility of admitting the consumption of only 50% of the prescribed diet and/or the maximum consumption of 4–5 kcal/kg weight of unplanned food in a single day between the days −2, −1 and +1 of each cycle. Adverse events were graded according to CTCAE version 5.0. Patients were excluded in the case of cancer progression or the unacceptable deterioration of nutritional status despite supplement administration.

### 2.5. Nutritional Status Evaluation

**Figure 1:**
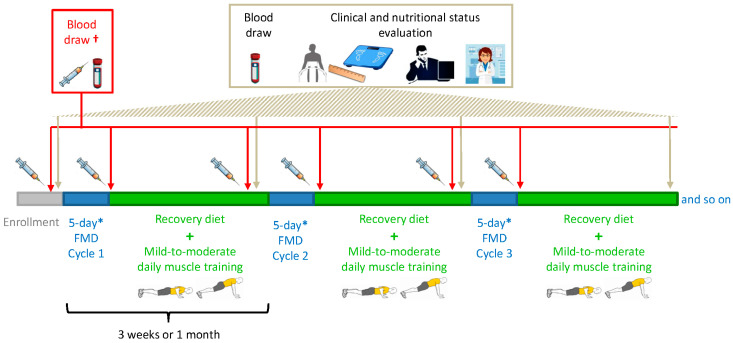
Intervention (fasting-mimicking diet (FMD)), clinical and nutritional status evaluation and blood sampling schedule. * Patients underwent FMD cycles of shorter duration (3- or 4-day FMD) if phase angle decreased to values between 5.2° and 5.0°. If the phase angle was <5°, the corresponding FMD cycle was not administered, and the patient was re-evaluated after 4 weeks and potentially recommended to take essential amino acids. † Only a small set of patients had an extra blood draw at day 6 of some of their FMD cycles.

The nutritional status was assessed before the initiation of each FMD cycle (Figure 1) in accordance with international guidelines. Anthropometric measures (weight, height and waist/abdomen/arm circumferences) were collected in the morning from fasting patients who were shoeless and wearing lightweight clothing. Weight and height were then used to calculate the BMI as weight (kg)/height (m^2^), which was further classified according to the World Health Organization’s age- and sex-adjusted criteria as undernourished if <18.5 kg/m^2^, normal weight if 18.5 to 24.9 kg/m^2^, overweight if 25 to 29.9 kg/m^2^ and obese if >30 kg/m^2^. 

The body composition (fat-free mass, fat mass, phase angle, extracellular mass-to-body cell mass ratio (ECM/BCM), total body water and intracellular water) was analyzed with a Single Frequency Bioimpedance Analyzer (BIA 101^®^, Akern, Florence, Italy) after at least 3 h of fasting. Bioelectrical impedance measurements were subsequently processed with the Bodygram Plus^®^ software (Akern, Florence, Italy). Handgrip strength was evaluated with the use of a dynamometer (T.K.K.5001 GRIP A Hand Grip Analogue Dynamometer, Takei, Japan).

### 2.6. Computed Tomography (CT) Scan-Based Body Composition Analysis

For CT scan analysis, both 1.25-mm and 5-mm slice thicknesses with a standard body kernel were used. The whole body was scanned from the lung apex to the pubic symphysis. Reconstructed axial images with both a 1.25-mm and a 5-mm slice thickness were analyzed using the software installed on the workstations of the IRCCS Ospedale Policlinico San Martino Radiology Department (Suite-Estensa 1.9-Ebit-Esaote Group Company, 2015). The third lumbar vertebra (L3), at the level in which both transverse processes are clearly visible, was the bony landmark for the estimation of total muscle area. In the case that only thoracic CT scans, but not abdominal CT scans, were available, the total muscle area was estimated at the level of the Louis angle (manubriosternal joint that lies at the level of the second costal cartilage). Estimation of the total muscle area was done at the baseline and after repeated FMD cycles as previously described [23].

### 2.7. ELISA Assays

Whole blood collection (by venous blood draws) followed by serum separation, as described elsewhere [2], for the determination of circulating levels of leptin, adiponectin, resistin, c-peptide (as a proxy for insulin production), IGF1, insulin-like growth factor-binding protein 1 (IGFBP1), IGFBP3, matrix metalloproteinase 8 (MMP8), MMP9, myeloperoxidase (MPO), tissue inhibitor of metalloproteinase 1 (TIMP1), TIMP2, MMP9/TIMP1 complex (M/T c), osteopontin (OPN), intercellular adhesion molecule 1 (ICAM1), vascular cell adhesion molecule 1 (VCAM1), sclerostin, interleukin-6 (IL-6) and C-reactive protein (hs-CRP) was routinely done after an overnight fast at enrollment (i.e., few days—typically one week—before the start of the first FMD cycle) and at the visit before each FMD cycle (i.e., after 2 to 3 weeks of re-feeding; Figure 1). 

In addition, for those patients who were available to return to our hospital immediately before re-feeding started (i.e., early on day 6 of one of their FMD cycles), serum was also obtained on that day (Figure 1). This allowed us to measure the serum levels of the factors mentioned above at the very end of the FMD period and, therefore, to assess the acute effects of the FMD. Patients’ sera were stored at −80 °C until their use. ELISA assays (from R&D Systems) for the quantification of growth factors, cytokines and adipokines were performed according to the instructions of the manufacturer.

### 2.8. Statistical Analysis

To evaluate changes in bioimpedance measurements as a function of time, a linear mixed-effects model was fitted. The model was adjusted for baseline fat mass to fat-free mass ratio as a normalization factor. *p*-values associated to the model coefficients were considered statistically significant if <0.05. The analysis was performed in the R environment using the lme4 package. The bioimpedance measurements considered were the fat-free mass (kg), fat mass (kg), weight (kg), handgrip strength (kg), phase angle (degrees), total body water (liters), intracellular water (liters) and ECM/BCM ratio. To reduce the sparsity of the data available across time, we analyzed the data until the 15th cycle, where data were available for at least 10% of patients. Statistical analyses of growth factors, adipokines and cyto/chemokines concentrations were performed using paired two-tailed Wilcoxon rank-sum test. Tests were performed in R environment and considered significant if *p*-value < 0.05.

## 3. Results

### 3.1. Patient Characteristics

**Figure 2:**
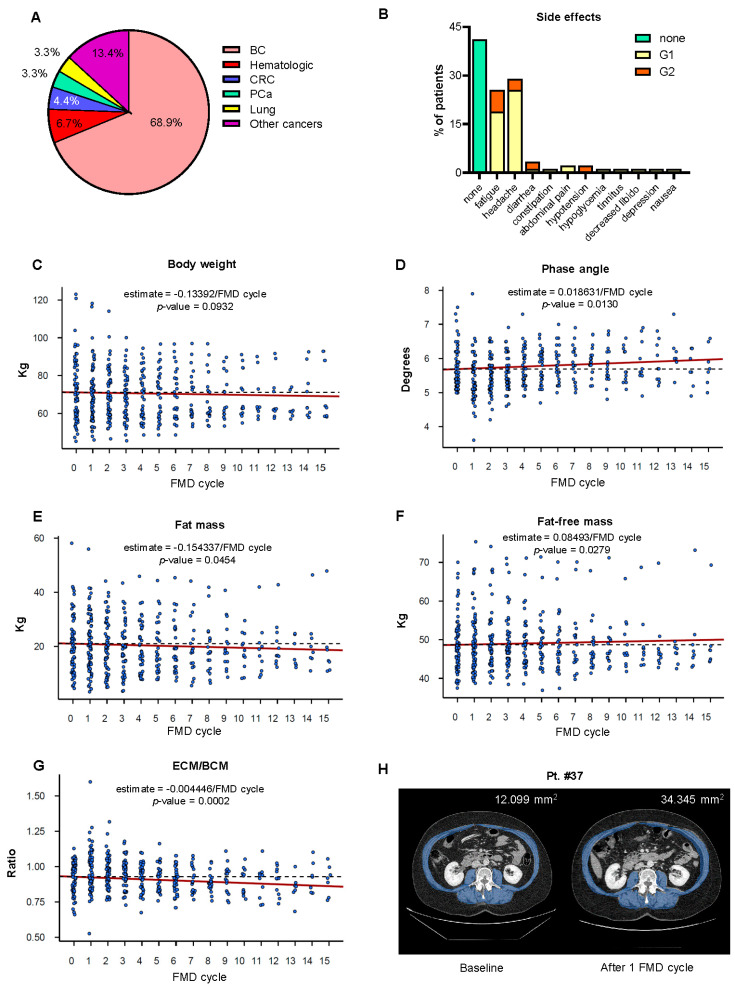
Effects of cyclic FMD on the nutritional parameters of cancer patients undergoing active anticancer treatments. (**A**) Pie chart representing the cancer diagnoses of the patients enrolled in the NCT03595540 clinical trial. (**B**) The adverse events of the FMD regime were graded according to CTCAE 5.0. (**C**–**G**) Body weight, phase angle, fat-free mass, fat mass and extracellular mass/body cell mass (ECM/BCM) ratio in patients with cancer receiving active medical treatment and cyclic FMD. To evaluate changes in these parameters, we fitted a linear mixed effects model taking into account absolute values as a function of time. (**H**) CT scan of Pt. #37 showing an increase in the total muscle area after one FMD cycle compared to the baseline. FMD: fasting-mimicking diet; BC: breast cancer; CRC: colorectal cancer; and PCa: prostate cancer.

From November 2017 to August 2020, 90 patients were enrolled. The patients’ characteristics are presented in Supplementary Table S1. At the time of enrollment, the average patient age was 50.4 ± 8 years (age range: 19–72), and 86% of the enrolled patients were females. The average BMI at enrollment was 25.9 ± 6.1 kg/m^2^ (range 19.0–44.1 kg/m^2^). The average phase angle at enrollment was 5.7 ± 0.6° (range 5.0°−7.5°). In our study, 18 different types of cancer diagnoses were represented, 5 of which were hematologic malignancies (Figure 2A). The most prevalent tumor type was breast cancer, with a total of 62 patients (68.89%). Among these, 44 were HER2-negative (HER2-), while 18 were HER2-positive (HER2+).

Thirty-six breast cancer patients had HR+ tumors, while 26 had HR- tumors. Four patients had colorectal cancer (4.44% of all patients), all of them in stage IV. Additional types of solid and hematologic malignancies that enrolled patients were being treated for included prostate cancer (*n* = 3), glioma (*n* = 2), melanoma (*n* = 2), ovarian cancer (*n* = 2), non-small-cell lung cancer (NSCLC; *n* = 2), pancreatic ductal adenocarcinoma (*n* = 2), anal cancer (*n* = 1), cervical cancer (*n* = 1), endometrial cancer (*n* = 1), small-cell lung cancer (SCLC; *n* = 1), bladder cancer (*n* = 1), multiple myeloma (*n* = 2), acute lymphoblastic leukemia (*n* = 1), chronic myeloid leukemia (*n* = 1), essential thrombocytosis (*n* = 1) and polycythemia vera (*n* = 1). A total of 12 patients with HR+ breast cancer, who were enrolled while on their adjuvant or neo-adjuvant chemotherapy, went on to receive monthly cycles of the FMD also while on their subsequent endocrine therapy.

### 3.2. Feasibility

A total of 81 patients (90%) completed at least 1 FMD cycle, whereas 65 (72.2%) completed the study with a range of 2 to 21 FMD cycles (average: 6.3 cycles per patient). A total of 16 patients (18%) were lost to follow-up in the period between the enrollment and the first re-evaluation (after the first FMD cycle). One patient (1%) died before starting the FMD cycle due to chemotherapy-related gastrointestinal complications. During the study, 11 patients (12%) were excluded because of cancer progression. All the patients who underwent at least one FMD cycle (*n* = 81) fulfilled our feasibility criteria. 

However, 13 of these patients reported interrupting earlier at least one of the assigned FMD cycles (i.e., by just undergoing 3 or 4 days of the FMD) by their own decision, with an average of 2.2 reduced cycles per patient among these 13 subjects. Overall, out of the total 489 FMD cycles prescribed to our patients, 28 of them (5.73%) were reduced in duration (i.e., 3–4 days instead of 5 days) by their own choice. The low palatability of some of the FMD components (in particular some soups and snacks) and the feeling of hunger and/or of weakness that was communicated by the patients were the main reported reasons for reducing the duration of the FMD cycles.

### 3.3. Safety

Overall, patients reported mild and transient (Grade 1–2; 35 patients (39%) with G1 and 12 patients (13%) with G2) adverse events according to CTCAE version 5.0. The most common FMD-emergent adverse events were headache (26 patients, 29%) and fatigue (23 patients, 26%), followed by diarrhea (3 patients, 3%), abdominal pain (2 patients, 2%), nausea (1 patient, 1%), constipation (1 patient, 1%), decreased libido (1 patient, 1%) and symptomatic hypoglycemia (1 patient, 1%) (Figure 2B). There were no Grade 3–5 adverse events related to FMD.

### 3.4. Nutritional Status and Body Composition of Patients Undergoing Cyclic FMD

The patients’ nutritional status was monitored on a monthly basis through the analysis of body composition using bioimpedance measurement. After every FMD cycle, the patients reported an average weight loss of 2–2.5 kg, which was typically re-gained during the re-feeding period between subsequent FMD cycles. A total of 27 patients (30%) showed a significant decrease in phase angle and fat free mass after one of their FMD cycles. In these cases, the subsequent FMD cycles were reduced to 3 or 4 days. Ten patients (11%) underwent a drop in their phase angle value below 5°. 

In these patients, oral amino acid supplements were administered, and the subsequent FMD cycle was postponed until the occurrence of complete recovery of the nutritional status. Four patients (4%) were excluded due to persistently low phase angle values at bioimpedance analysis. Serial bioimpedance measurements showed a statically significant increase in phase angle and in fat-free mass, as well as a consistent decrease in the ratio between ECM and BCM (Figure 2D,F,G). The total body water and intracellular water both increased, (Figure S1A,B), while the fat mass was reduced (Figure 2E). Handgrip strength and body weight did not change throughout the study (Figure 2C and Figure S1C). 

Abdominal and thoracic CT scans were used to monitor body composition in those patients who were prescribed these imaging studies for disease restaging or for monitoring response to treatment (Pts #1, #3, #37, #40, #48 and #89). Muscle and subcutaneous fat tissue representation in patients #37, #40, #48 and #89 are presented in Table 1 (Pts #1 and #3 were already shown in Caffa et al. [2]), while Figure 2H shows abdominal CT scans of Pt #37 at baseline and after 1 FMD cycle. Overall, the muscle and fat tissue representation remained stable or increased during the study period in these patients.

### 3.5. Effect of the FMD on Circulating Growth Factors, Adipokines and Cyto/Chemokines

**Figure 3:**
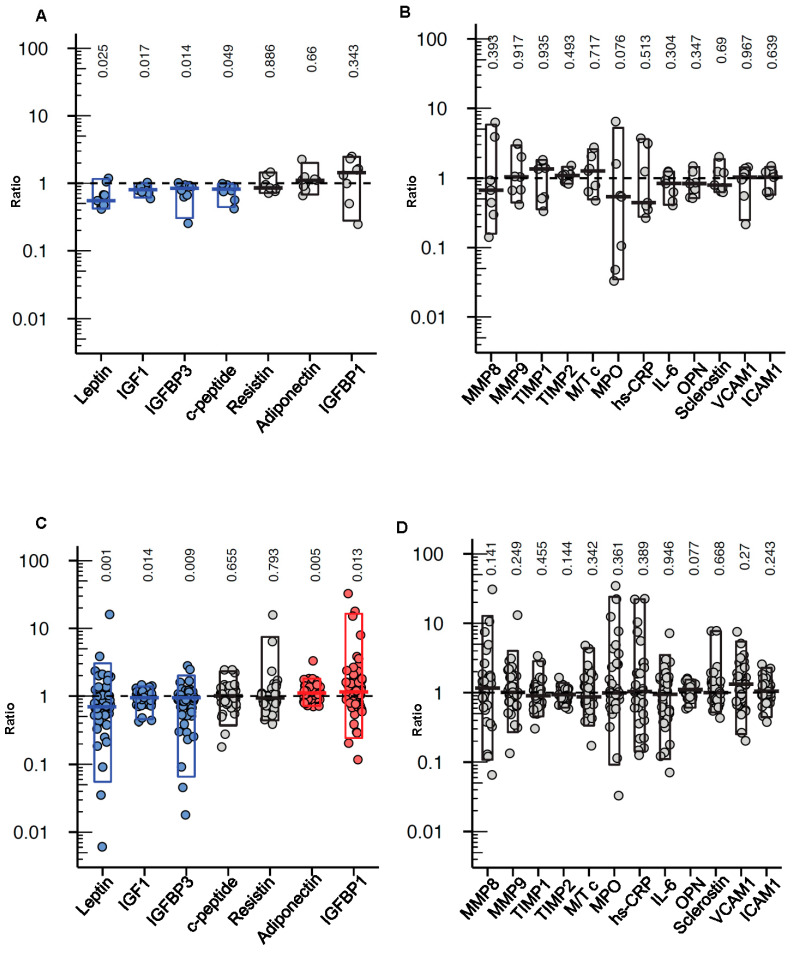
Effects of cyclic FMD on circulating growth factors, adipokines, metalloproteinases and cyto/chemokines in cancer patients. Serum leptin, adiponectin, resistin, c-peptide, IGF1, IGFBP1, IGFBP3 ((**A**), *n* = 9), MMP8, MMP9, MPO, TIMP1, TIMP2, MMP9/TIMP1 complex (M/T c), OPN, ICAM1, VCAM1 and hs-CRP ((**B**), *n* = 7) before the start of a FMD cycle and immediately after it (on day 6, before re-feeding started); and before the start of the first FMD cycle (baseline) and a few days before the start of the second FMD (i.e., after 2 to 3 weeks of re-feeding; (**C**), *n* = 62; (**D**), *n* = 34) were measured by ELISA. The results are presented as fold changes vs. the pre-FMD values and were analyzed by paired two-tailed Wilcoxson rank-sum test, with the corresponding *p*-values shown on the top of each graph.

When measuring the levels of growth factors, adipokines and cyto/chemokines in serum samples obtained few days before and immediately after a FMD cycle (on day 6, immediately before re-feeding; to detect the “acute” effect of the FMD), we found the c-peptide (a proxy for insulin production), IGF1, leptin and IGFBP3 levels to be reduced at the end of the FMD period (Figure 3A), while no effect of the diet on IGFBP1, resistin, adiponectin or any of the cyto/chemokines and adhesion molecules tested was documented (Figure 3A,B). 

We also analyzed serum samples that were obtained before the start of the first FMD cycle (baseline) and after 2 to 3 weeks of re-feeding after the first FMD to detect potential long-lasting/carry-over effects of the diet. In the samples collected two to three weeks after the end of the first FMD cycle, the leptin, IGF1 and IGFBP3 levels were found to be still lower as compared to the baseline levels, while the adiponectin and IGFBP1 levels were higher (Figure 3C). Again, no significant effect of the FMD on any of the cyto/chemokines tested was found (Figure 3D).

## 4. Discussion

In the present phase I/II clinical trial, we showed that periodic FMD cycles were feasible and safe in cancer patients at low nutritional risk and that they did not negatively affect the patient body composition and nutritional status. Unlike previously published studies of fasting/FMD in cancer patients, which only enrolled patients receiving concomitant chemotherapy [12,13,14,15,16,17], in our trial, we accrued patients who were treated with different types of therapies, including chemotherapy (*n* = 46), endocrine therapies for breast cancer (*n* = 36), for prostate cancer (*n* = 2) and for endometrial cancer (*n* = 1), TKIs (*n* = 3), CDK4/6 inhibitors (*n* = 4), proteasome inhibitors (*n* = 2), immune check point inhibitors (*n* = 3), immunomodulators (*n* = 2), other biologicals (*n* = 13), radiotherapy (*n* = 7), glucocorticoid (*n* = 2) or with Bacillus Calmette–Guerin therapy (for superficial bladder cancer (*n* = 1)). 

In patients who received treatments other than chemotherapy, the FMD was also found to be largely feasible and essentially safe. This broad feasibility and safety of a FMD regime in patients with different types of cancer and undergoing diverse types of treatment is an important element of novelty of our study, considering that potential benefits of fasting/modified fasting regimes in combination with several non-chemotherapy anticancer treatments have been proposed (including TKIs, endocrine therapies for breast cancer, radiotherapy and immune checkpoint inhibitors) [1,2,3,4,24].

It is noteworthy that, in our study, we observed a better adherence to the FMD as compared with the Dutch DIRECT study, which utilized a similar dietary tool [13]. In the first place, we ascribe this difference to the fact that the FMD we utilized is less calorie restricted than the one used by De Groot et al. (i.e., the FMD we used provides 1100 kcal on day 1 and ~700 kcal on days 2–5, while the FMD used in the DIRECT study provides ~1200 kcal on day 1 and ~200 kcal on days 2–4). In addition, we believe that the involvement of a nutritionist (who was frequently in touch with the patients during the FMD days, addressing their problems/concerns and motivating them to pursue the diet) in our trial, likely also played a role in ensuring a good compliance with the FMD.

In addition to overall good feasibility and safety profile, the bioimpedance measurements we performed throughout this study showed favorable modifications in terms of the phase angle, fat-free mass, fat mass and BCM (in the context of a substantial stability of body weight). We found a consistent decrease in the ECM/BCM ratio, which directly reflects the proportions between intra- and extracellular spaces and is one of the most sensitive indexes of malnutrition [25]. The ECM includes all metabolically inactive tissues of the body, while the BCM includes all the metabolically active tissues; therefore, the ECM/BCM ratio has been widely used as a tool in the evaluation of body composition and nutrition status in many conditions, including cancer [26]. Our observation is important given that malnutrition is commonly observed in numerous cancer patients, it is significantly associated with the risk of disease complication during therapy leading to longer patient hospitalizations, and potentially negatively affecting treatment outcomes. We also observed an increase in the phase angle, which could be of relevance given that a low phase angle may indicate deterioration of the cell membrane in healthy tissues [27], which, in advanced cancer patients (i.e., metastatic disease), may result in a shorter overall survival. 

Indeed, a systematic review of nutritional studies in cancer patients related a low phase angle with worse nutrition status, evaluated by BMI, serum albumin level, transferrin and fat-free mass, and the researchers found a significant positive association between the phase angle and overall survival [28]. The preservation of muscle mass and function was also confirmed through the estimation of psoas and total muscle areas in CT scans from a subset of patients. These results differ from those by Zorn and colleagues, who found that repeated modified fasting cycles resulted in a decrease in the phase angle value and in the body cell mass in women treated with chemotherapy for gynecological cancers [17]. We believe that these differences could be attributable to several reasons: (i) the FMD used in our study had a different composition and was less calorie-restricted than the modified fasting protocol prescribed by Zorn and colleagues (providing only 400–600 kcal/day for four days); (ii) in the time interval between FMD cycles, we prescribed a personalized diet that was enriched in total calories and in protein content; in addition, patients presenting with a more marked loss in lean body mass were also prescribed amino acid supplements; and (iii) in our study, but not in the trial by Zorn and coworkers, patients were recommended to exercise daily according to a dedicated training program to enhance their muscle anabolism in the interval between consecutive FMD cycles. 

Given the complexity of the nutritional intervention that we implemented, it is unknown which one of these factors was the most relevant for the observed beneficial effects on body composition. Ultimately, it is possible that, at different degrees, all of them contributed to such effects and that the type of multimodal intervention we implemented may be key to ensuring a safe introduction of fasting-based dietary regimes in oncology. The observation that cyclic administrations of the FMD led to a reduction in adipose tissue is in line with the reduction in leptin levels that we found. Taking into account that excess body adiposity exerts multiple pro-oncogenic effects, that it is a risk factor for many common adult cancers and that it is associated with an impaired response to different types of anticancer treatments, including neoadjuvant chemotherapy and endocrine therapy plus CDK4/6 inhibitors for breast cancer [29,30,31], the reported effects of the FMD on cancer patients’ fat mass have a strong potential value and lend further support to its evaluation in future clinical studies.

In line with our previously published data in a subset of patients with HR+ breast cancer (*n* = 24), metabolic evaluations performed in the whole patient cohort showed a significant reduction of circulating levels of c-peptide, IGF1 and leptin after the FMD. IGF1 bioavailability was also decreased by the FMD, as indicated by reduced IGFBP3 and increased IGFBP1 levels [32]. These results are especially relevant in the light of the fact that reduced blood levels of insulin, IGF1 and leptin were previously shown to enhance the activity of chemotherapy, endocrine therapies and inhibitors of the PI3K-mTOR pathway [2,4,7,33]. Even more interestingly, we observed that the leptin, IGF1 and IGFBP3 levels remained lower than those found at the baseline two-to-three weeks after the end of the FMD period, while adiponectin and IGFBP1 stayed higher. This indicates that some metabolic effects of the FMD persist for extended periods, possibly contributing to create long-lasting unfavorable conditions for tumor growth [2].

This clinical trial was not designed to assess the clinical efficacy of the FMD regime in terms of its anticancer activity. Therefore, no conclusion in that respect can be drawn from this study. However, the outcomes in certain patient groups are worth discussing. Specifically, seven patients from this trial were treated with endocrine therapy with or without CDK4/6 inhibitors for metastatic HR+/HER2- breast cancer. We recently reported that, in mice, adding periodic fasting or a FMD to endocrine therapy plus palbociclib potentiated its activity and delayed acquired resistance to this combinatorial therapy [2]. 

Clinical outcomes in these patients were, indeed, promising. Pt. #1 received eight FMD cycles while on treatment with fulvestrant plus palbociclib in the second-line setting, only progressing after 32 months (progression-free survival in this setting is on average of 9.5 months) [34]. Pt. #81 received seven monthly FMD cycles while on first-line palliative treatment with fulvestrant plus abemaciclib. She currently remains on trial and has had a clinically controlled disease for a total of 18 months. 

Regarding to patients treated with hormonal therapy, Pt. #3 and Pt. #7 received 13 and 12 FMD cycles, respectively, while on first-line treatment with an aromatase inhibitor. Pt. #3 relapsed after 24 months from enrollment, while Pt. #7 has had now stable disease for 29 months. Results in the latter two patients are also remarkable since the reported mean duration of response to aromatase inhibitors at this disease stage is between 12 and 18 months [34]. 

Overall, together with the outcomes from the other patients with advanced disease or with hematologic malignancies enrolled in our trial (Supplementary Table S1), these findings are consistent with those of previous studies [13,16], indicating that periodic cycles of fasting/FMD does not prevent the ability of different types of anticancer agents to exert their activity. Rather, in selected cases, such as in patients with breast cancer undergoing endocrine therapy, the clinical outcomes with the addition of FMD cycles were interesting and potentially worth studying further in dedicated clinical trials also in light of the strong preclinical evidence of efficacy [2,7].

## 5. Conclusions

Our trial showed that, when combined with dietary and muscle training instructions to promote weight and lean body mass re-gain in the periods between FMD cycles, even multiple administrations of this dietary regime were safe in cancer patients at low nutritional risk. The FMD decreased fat mass and effectively lowered the circulating insulin, IGF1 and leptin. Future studies should focus on the efficacy of FMDs in specific clinical settings, such as in patients with advanced HR+/HER2- breast cancer undergoing endocrine therapy.

## Figures and Tables

**Table 1:** CT-scan based analysis of body composition in patient #37, #40, #48 and #89.

Pt. #	FMD Cycles	Psoas Muscle Area (mm^2^)	Total Muscle Area (mm^2^)	Subcutaneous Fat Area (mm^2^)
**37**	Baseline	670	12,099	29,380
	After 3 FMD cycles	680	34,345	30,305
**40**	Baseline	226	3634	3826
	After 1 FMD cycle	222	3341	7249
**48**	Baseline	1712	13,007	12,674
	After 4 FMD cycles	1933	13,733	13,500
**89**	Baseline	821	9434	3120
	After 6 FMD cycles	817	8806	3210

FMD: fasting-mimicking diet; and Pt. #: patient number.

## Data Availability

The data presented in this study are available on request from the corresponding author.

## Supplementary Materials

The following data are available online at https://www.mdpi.com/article/10.3390/cancers13164013/s1. Table S1: Patient characteristics, adverse events and best response; Figure S1: Effects of cyclic FMD on the nutritional parameters of cancer patients undergoing active anticancer treatments.
