# Supplementary material for: Safety and Feasibility of Fasting-Mimicking Diet and Effects on Nutritional Status and Circulating Metabolic and Inflammatory Factors in Cancer Patients Undergoing Active Treatment
Source: Cancers (Basel). 2021 Aug 9;13(16):4013. doi: 10.3390/cancers13164013 (PMC8391327; doi:10.3390/cancers13164013)
Supplement: Supplementary file 1 [file cancers-13-04013-s001.zip › cancers-1317183-supplementary.pptx]

## Slide 1
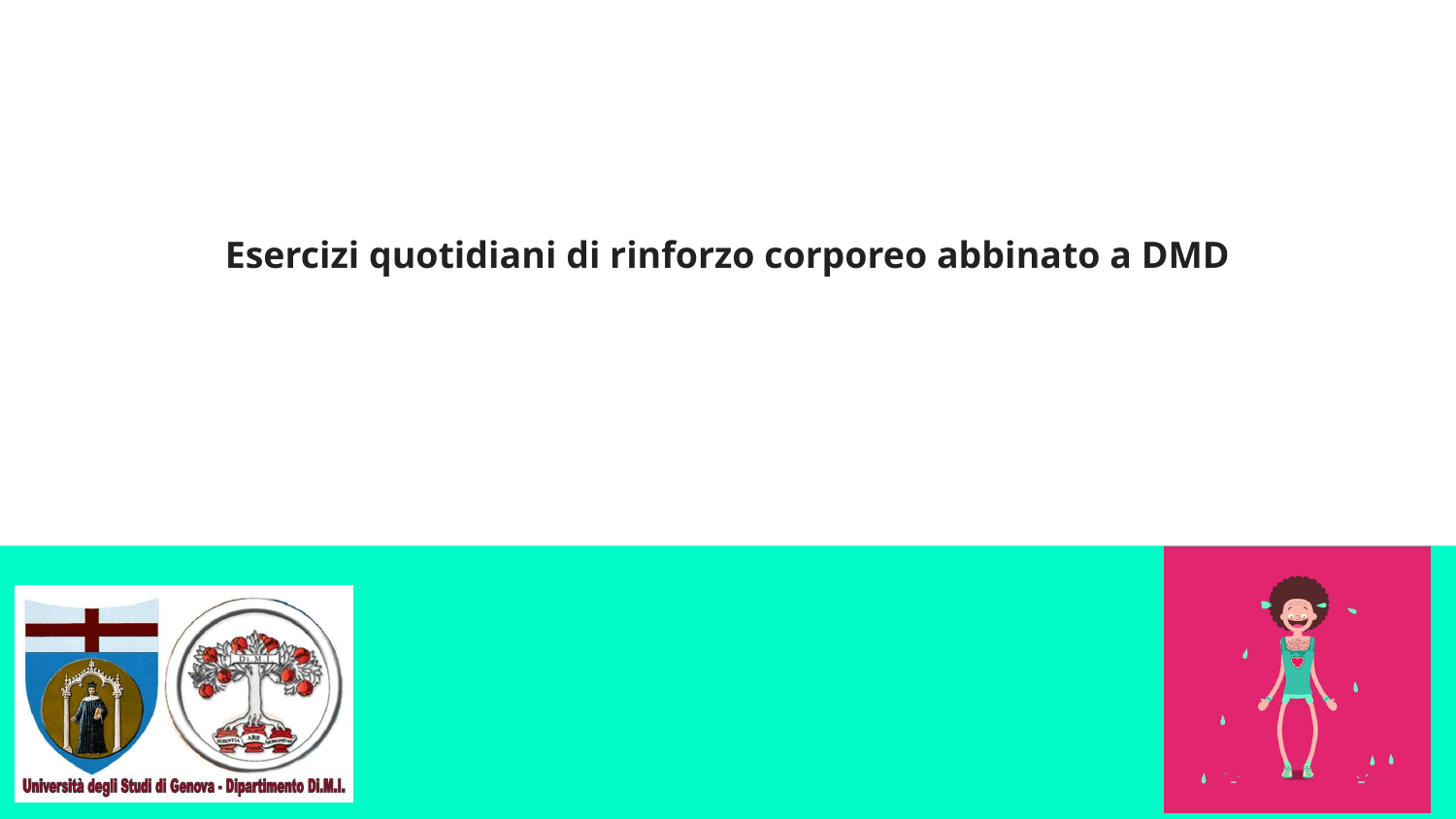

# Esercizi quotidiani di rinforzo corporeo abbinato a DMD

## Slide 2
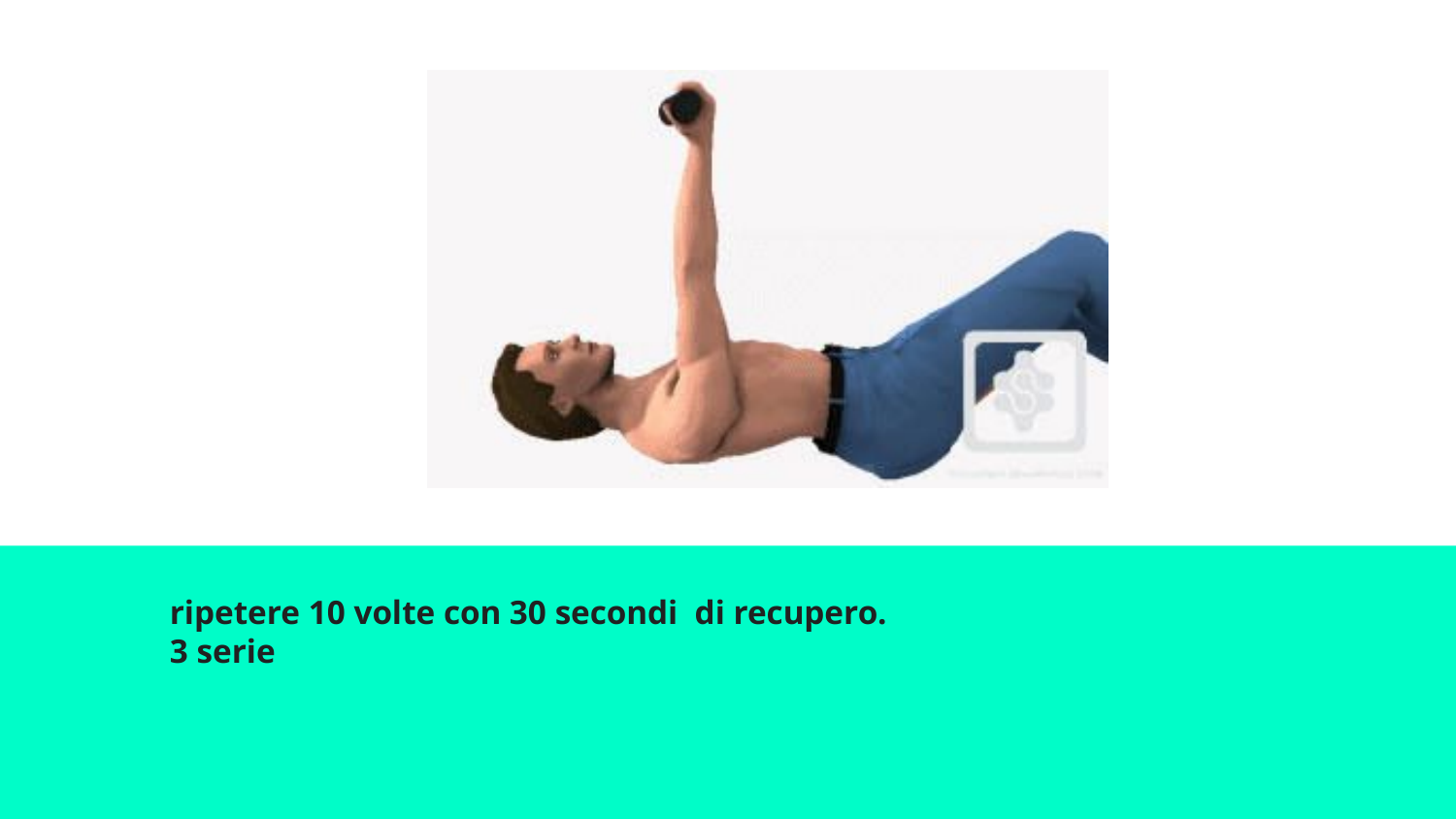

ripetere 10 volte con 30 secondi di recupero.
3 serie

## Slide 3
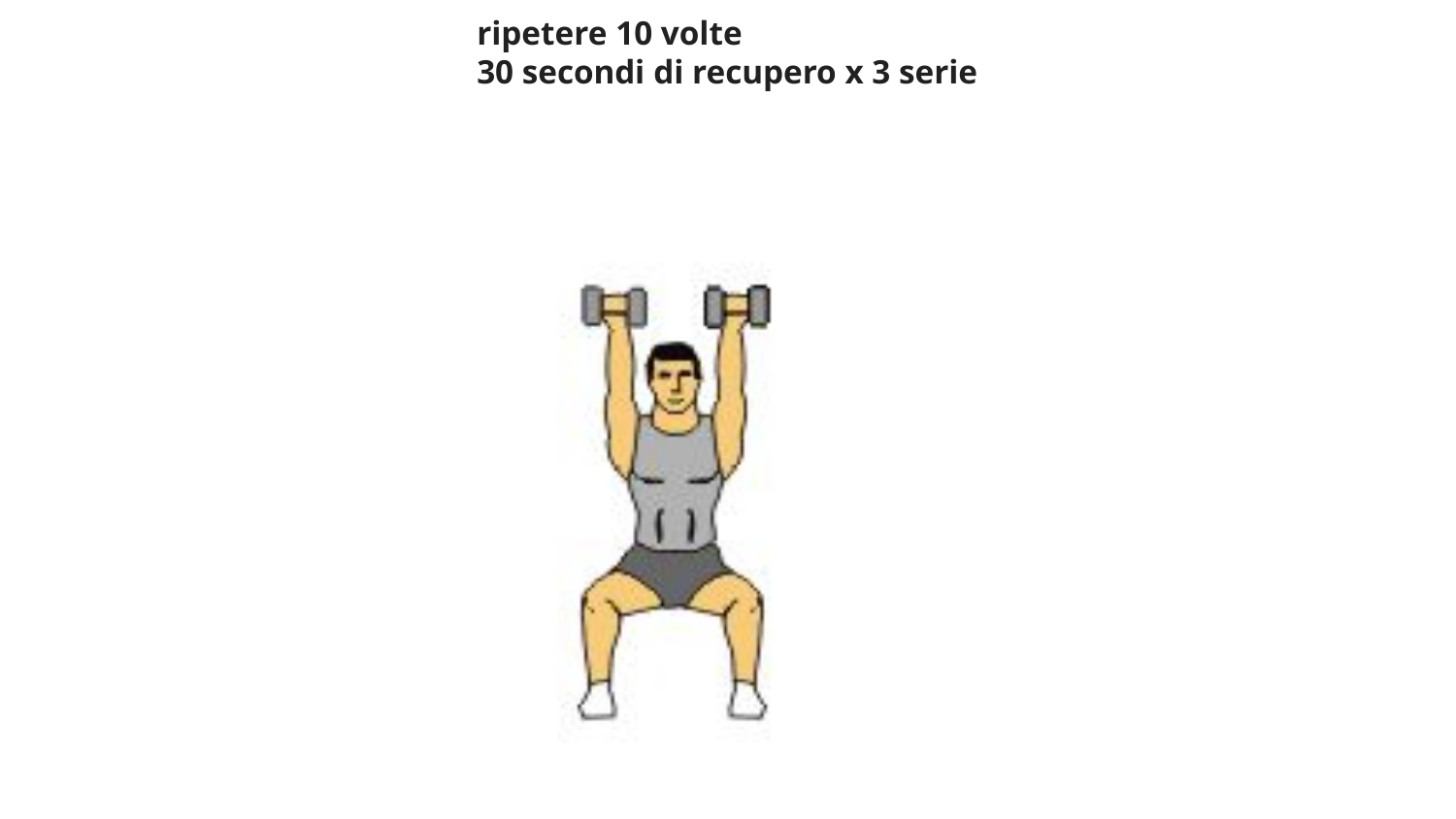

# ripetere 10 volte
30 secondi di recupero x 3 serie

## Slide 4
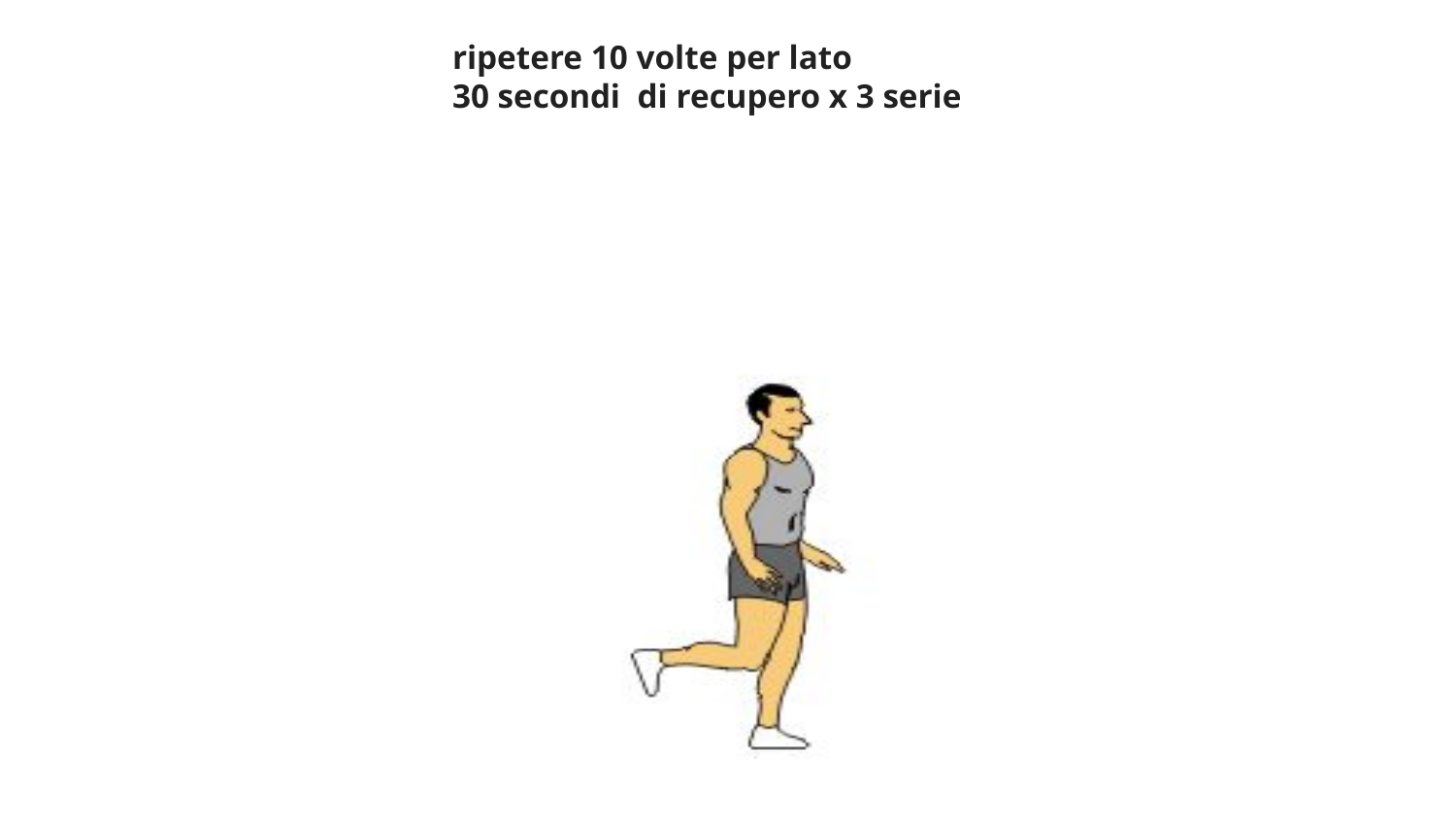

# ripetere 10 volte per lato
30 secondi di recupero x 3 serie

## Slide 5
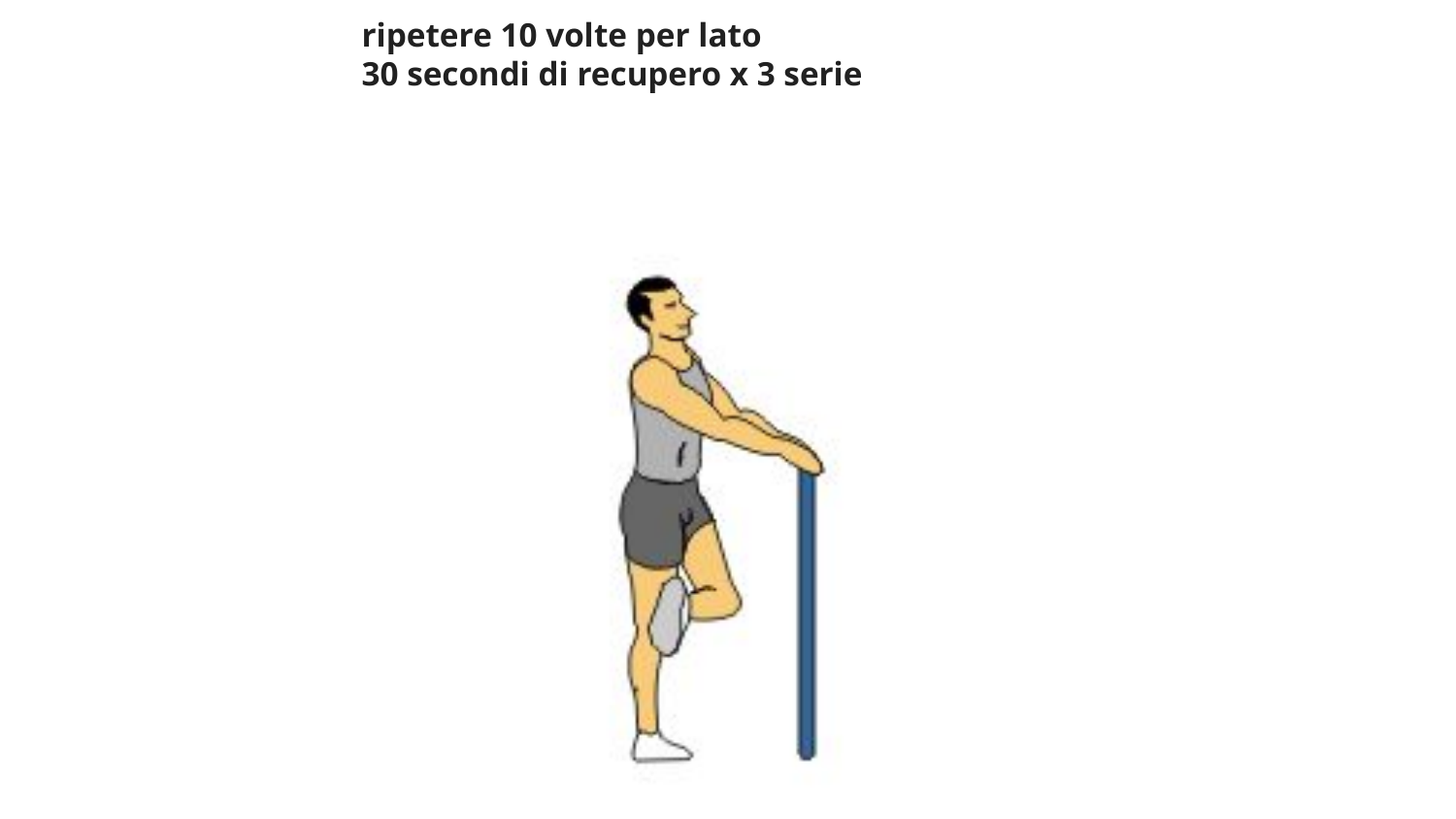

# ripetere 10 volte per lato
30 secondi di recupero x 3 serie

## Slide 6
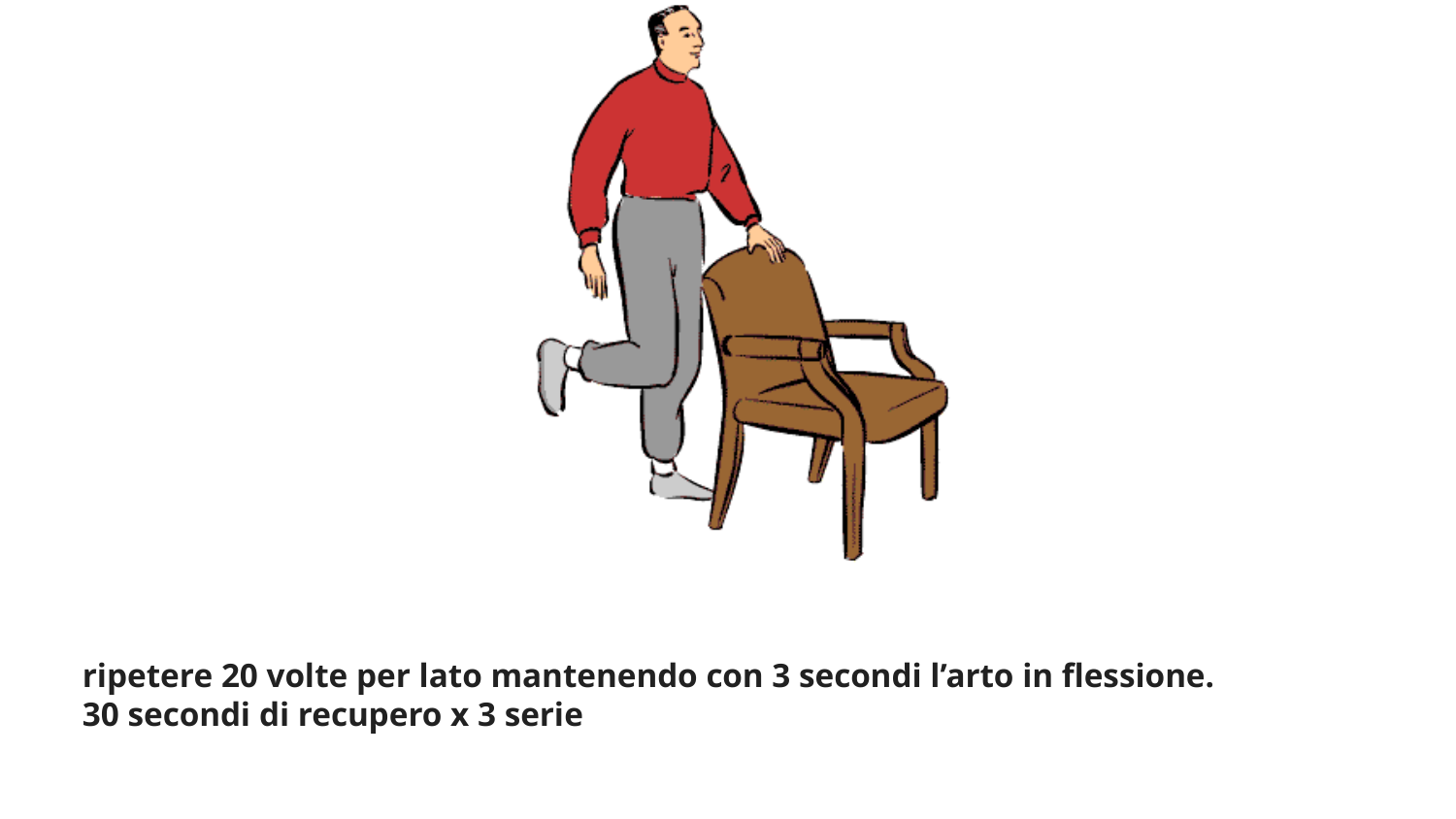

ripetere 20 volte per lato mantenendo con 3 secondi l’arto in flessione.
30 secondi di recupero x 3 serie

## Slide 7
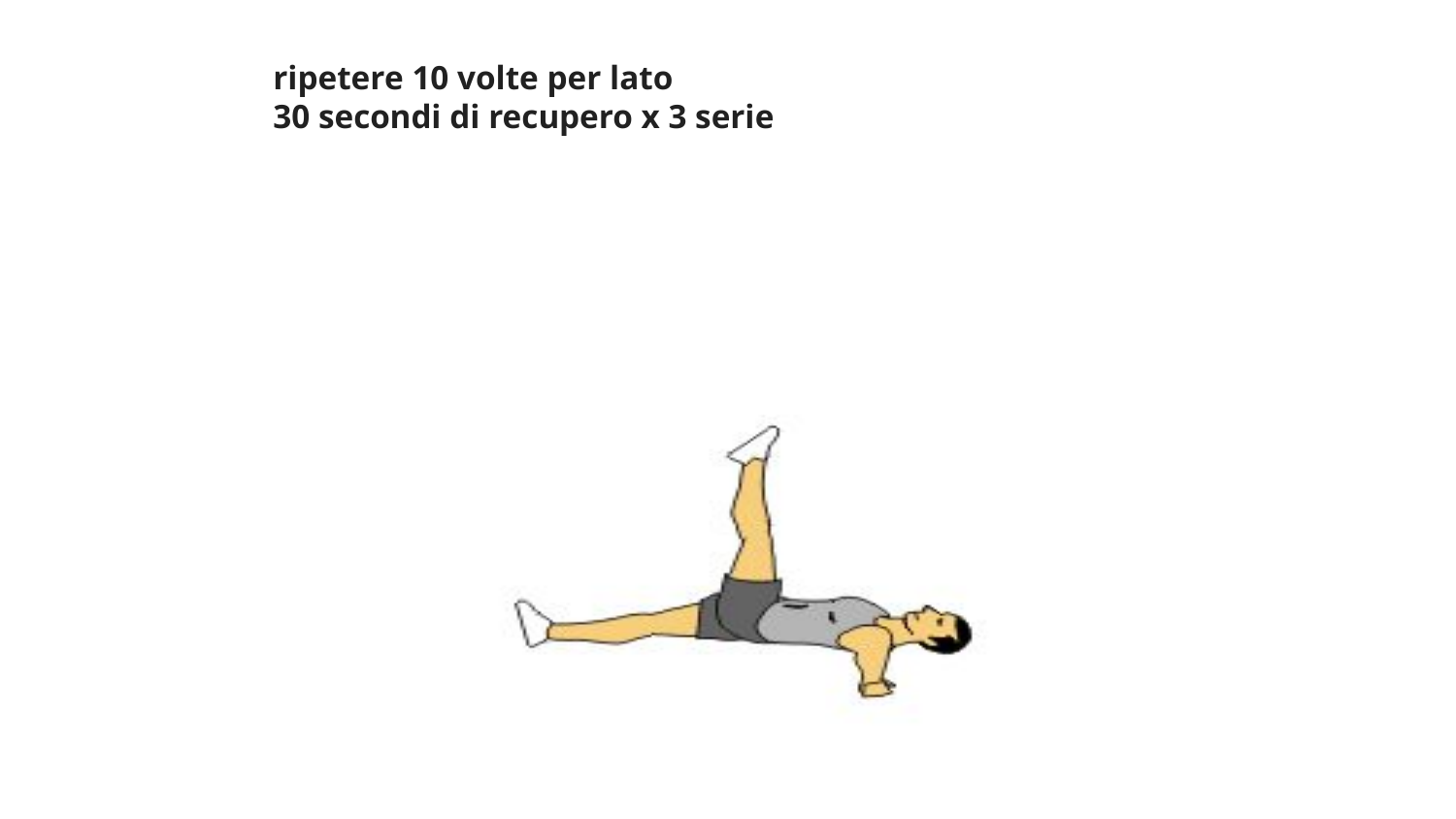

# ripetere 10 volte per lato
30 secondi di recupero x 3 serie

## Slide 8
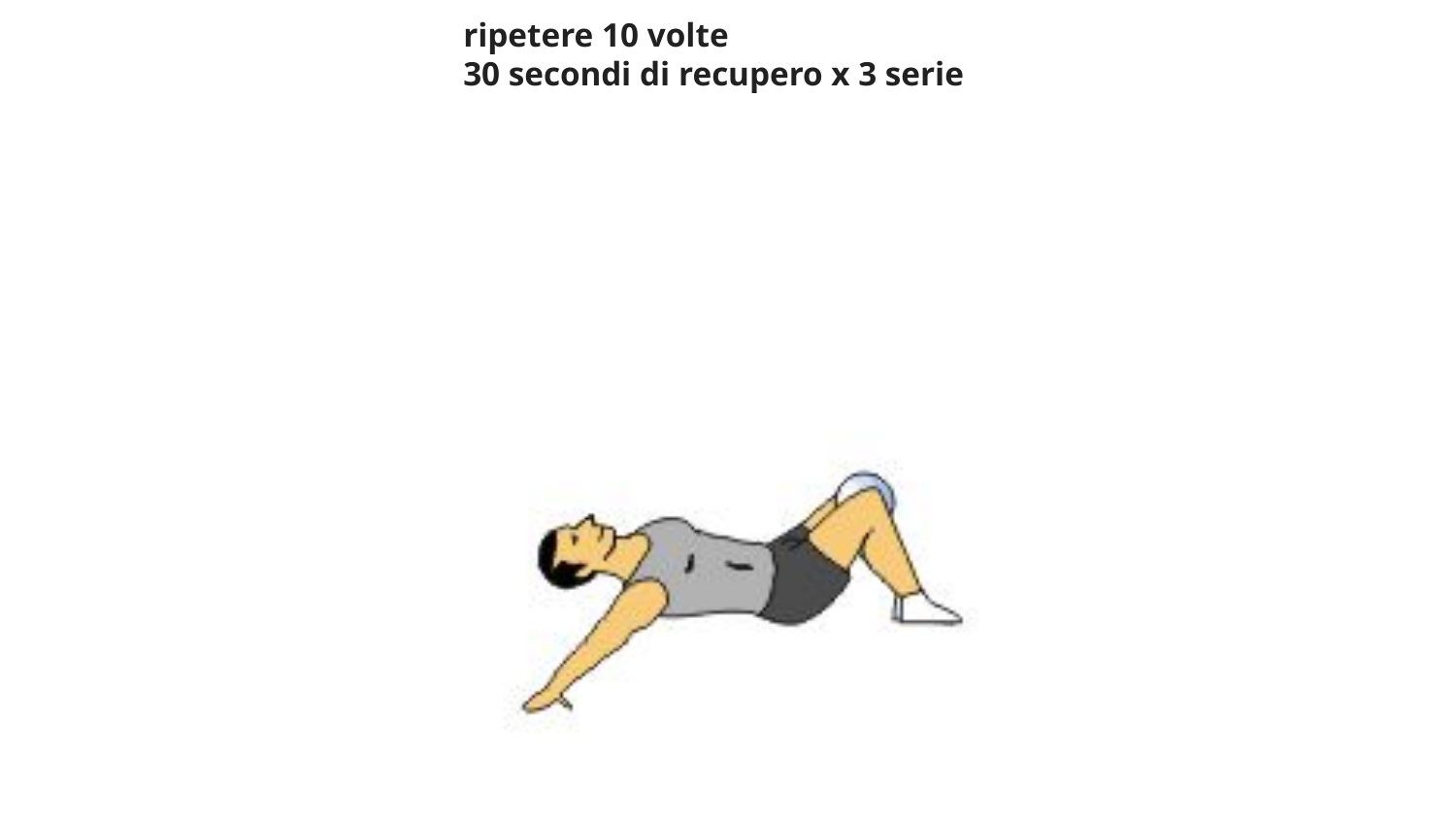

# ripetere 10 volte
30 secondi di recupero x 3 serie

## Slide 9
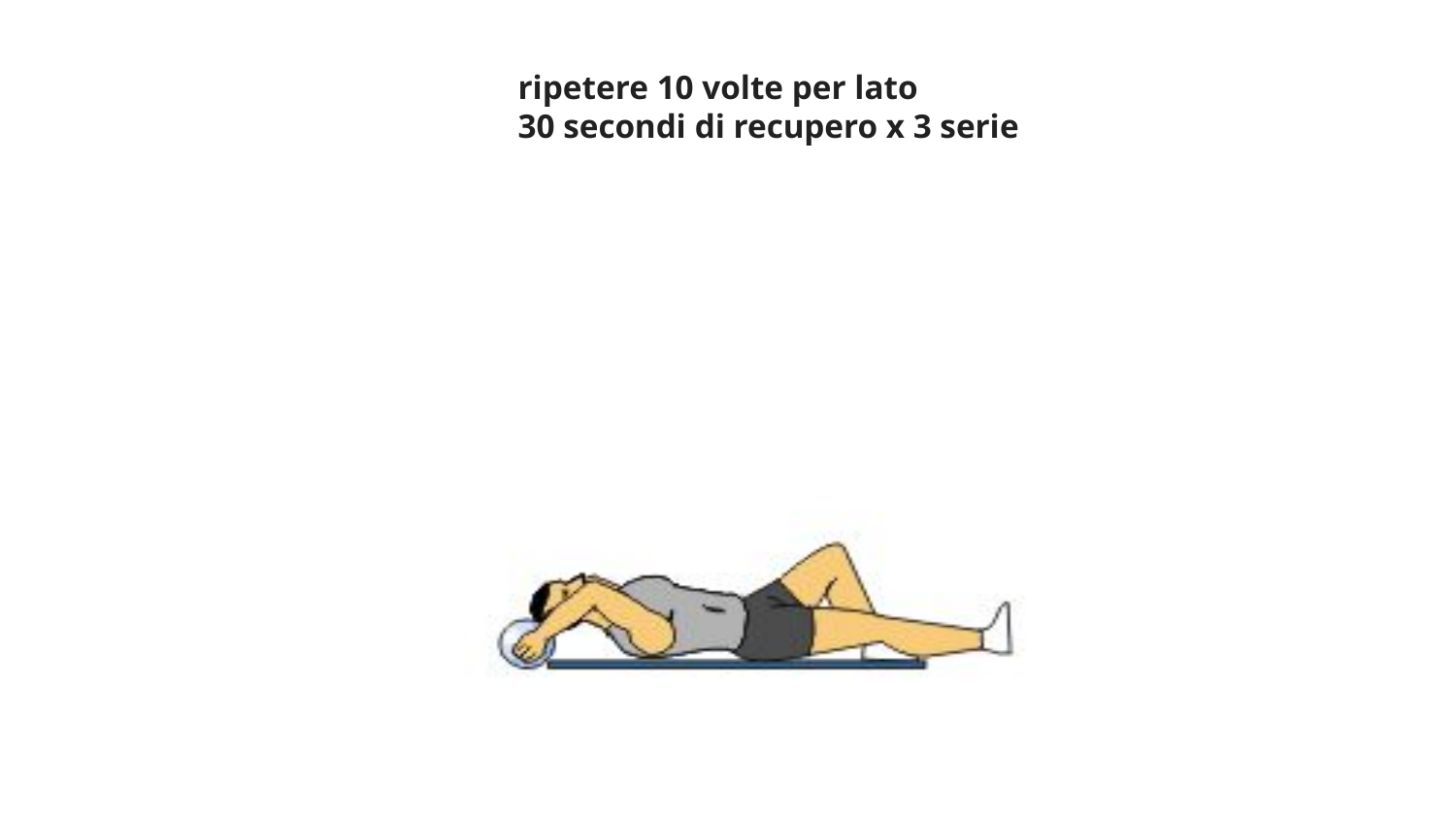

# ripetere 10 volte per lato
30 secondi di recupero x 3 serie

## Slide 10
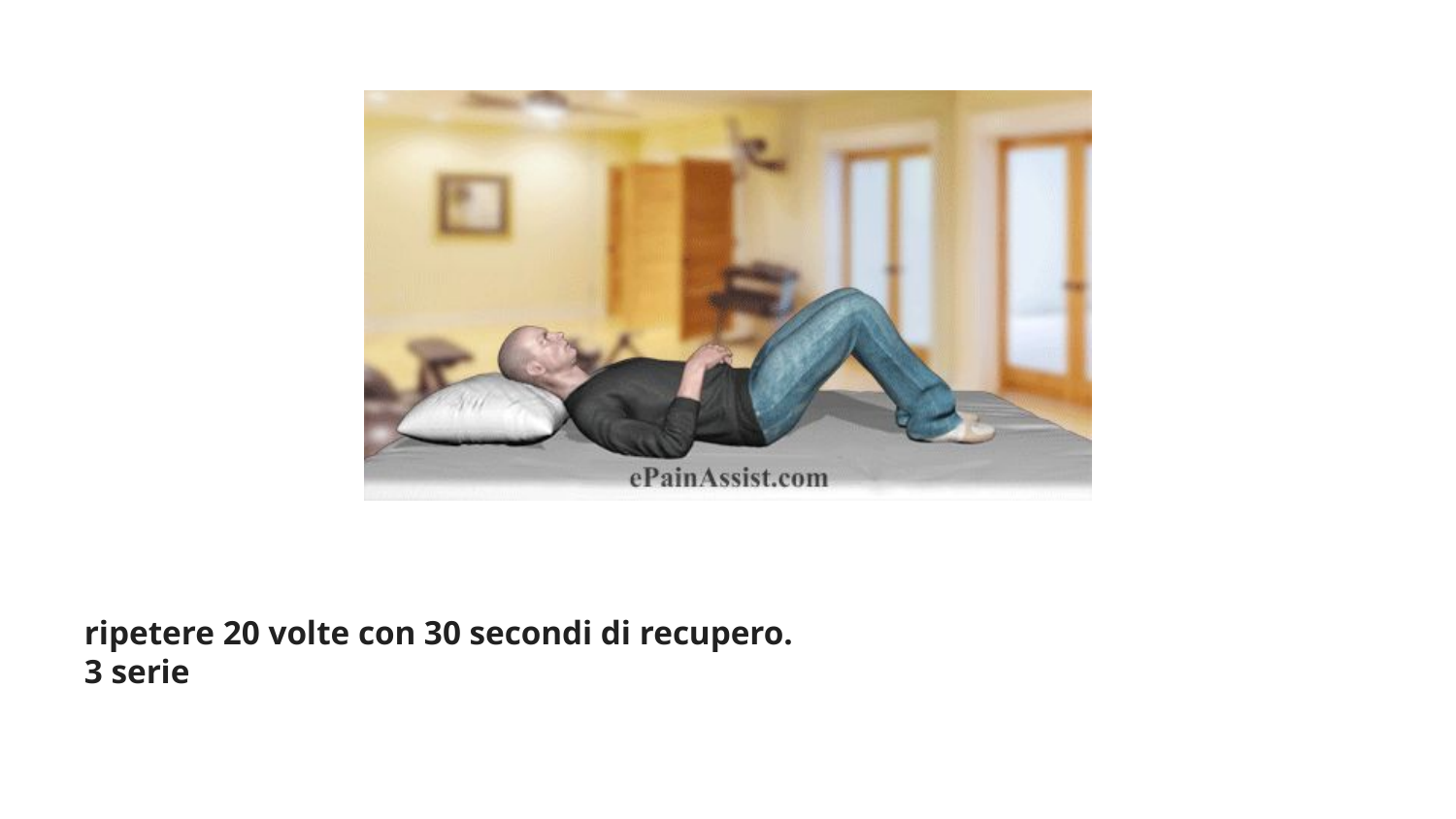

ripetere 20 volte con 30 secondi di recupero.
3 serie

## Slide 11
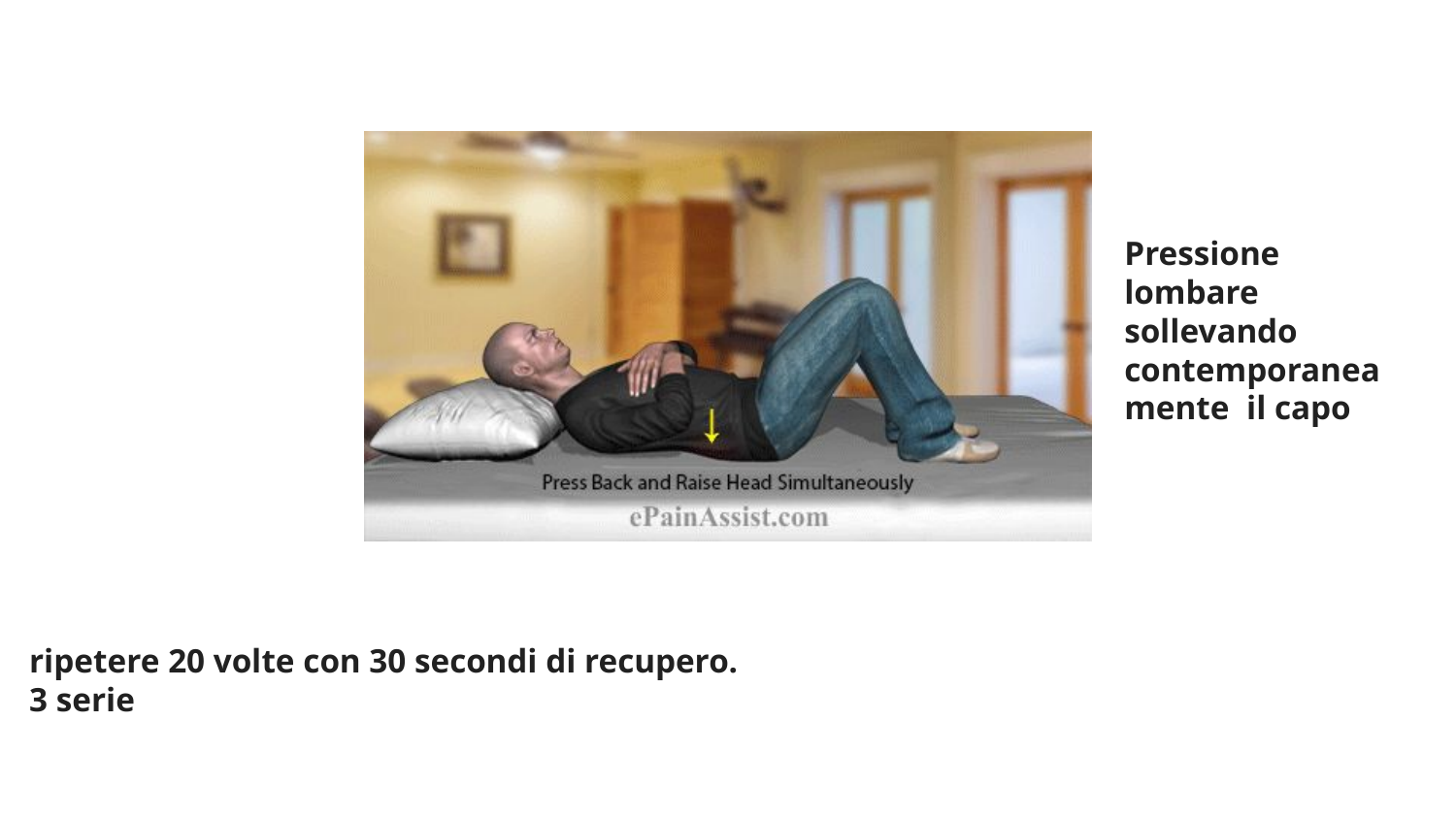

Pressione lombare sollevando contemporaneamente il capo
ripetere 20 volte con 30 secondi di recupero.
3 serie

## Slide 12
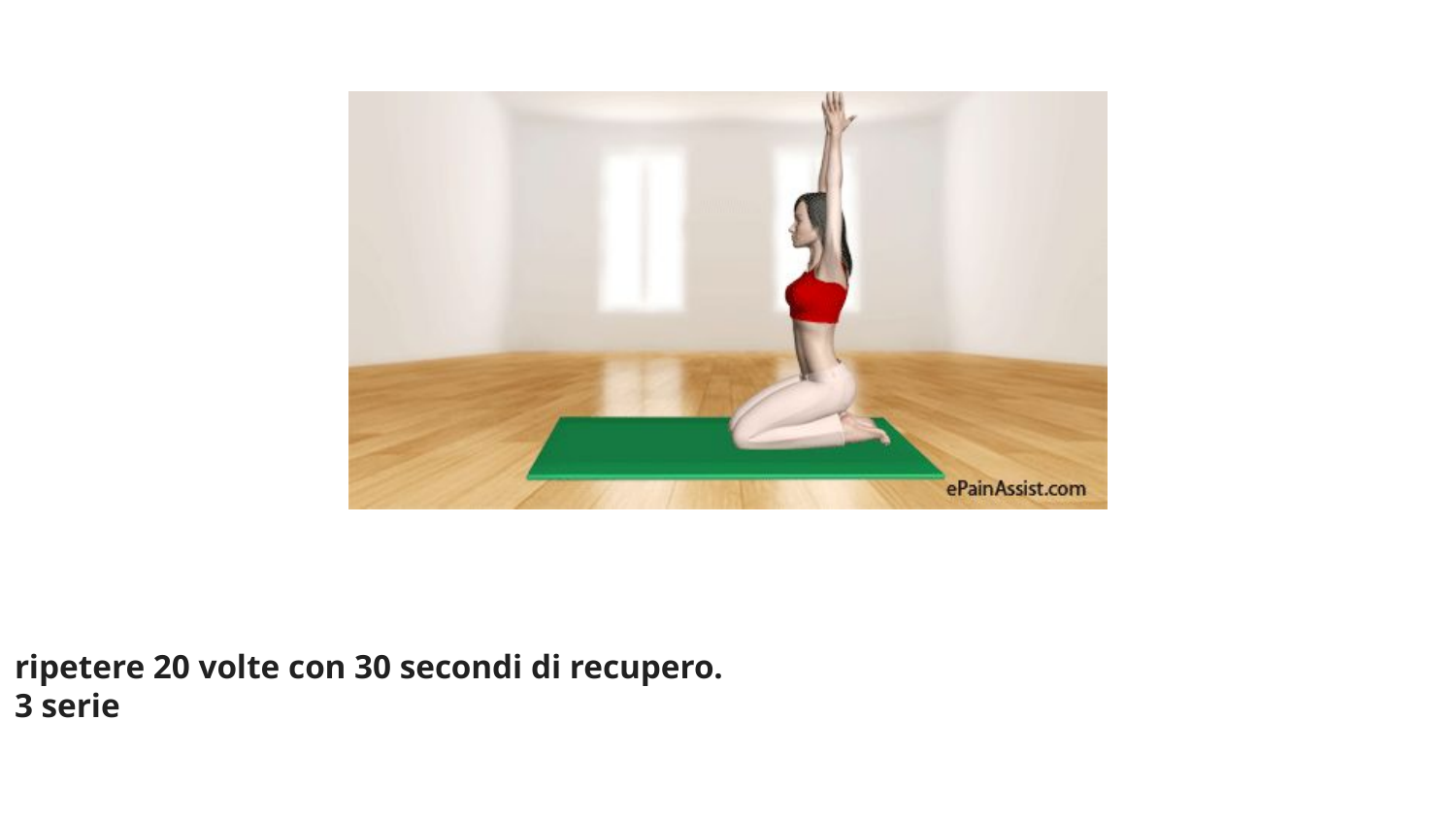

ripetere 20 volte con 30 secondi di recupero.
3 serie

## Slide 13
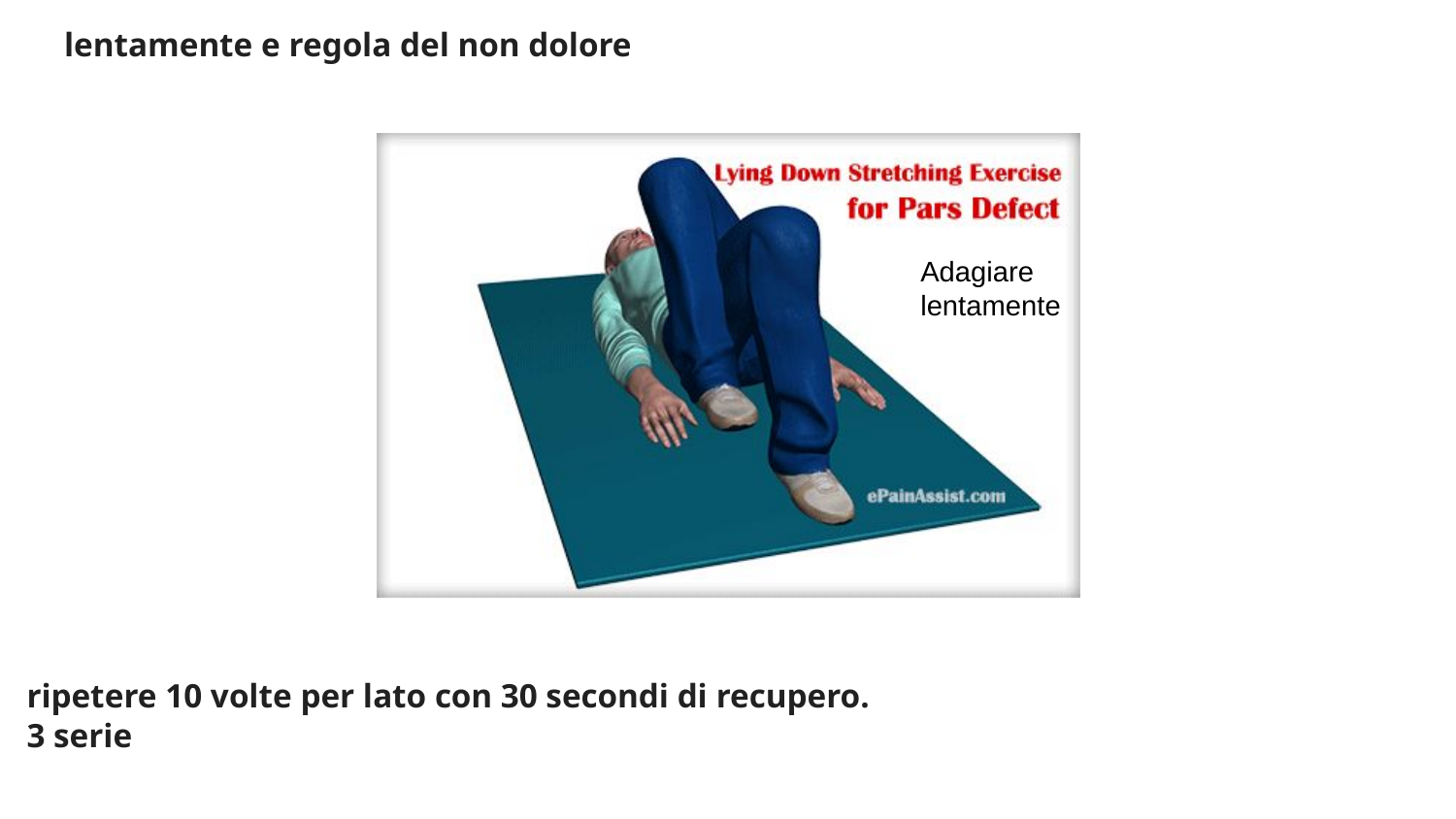

# lentamente e regola del non dolore
Adagiare lentamente
ripetere 10 volte per lato con 30 secondi di recupero.
3 serie

## Slide 14
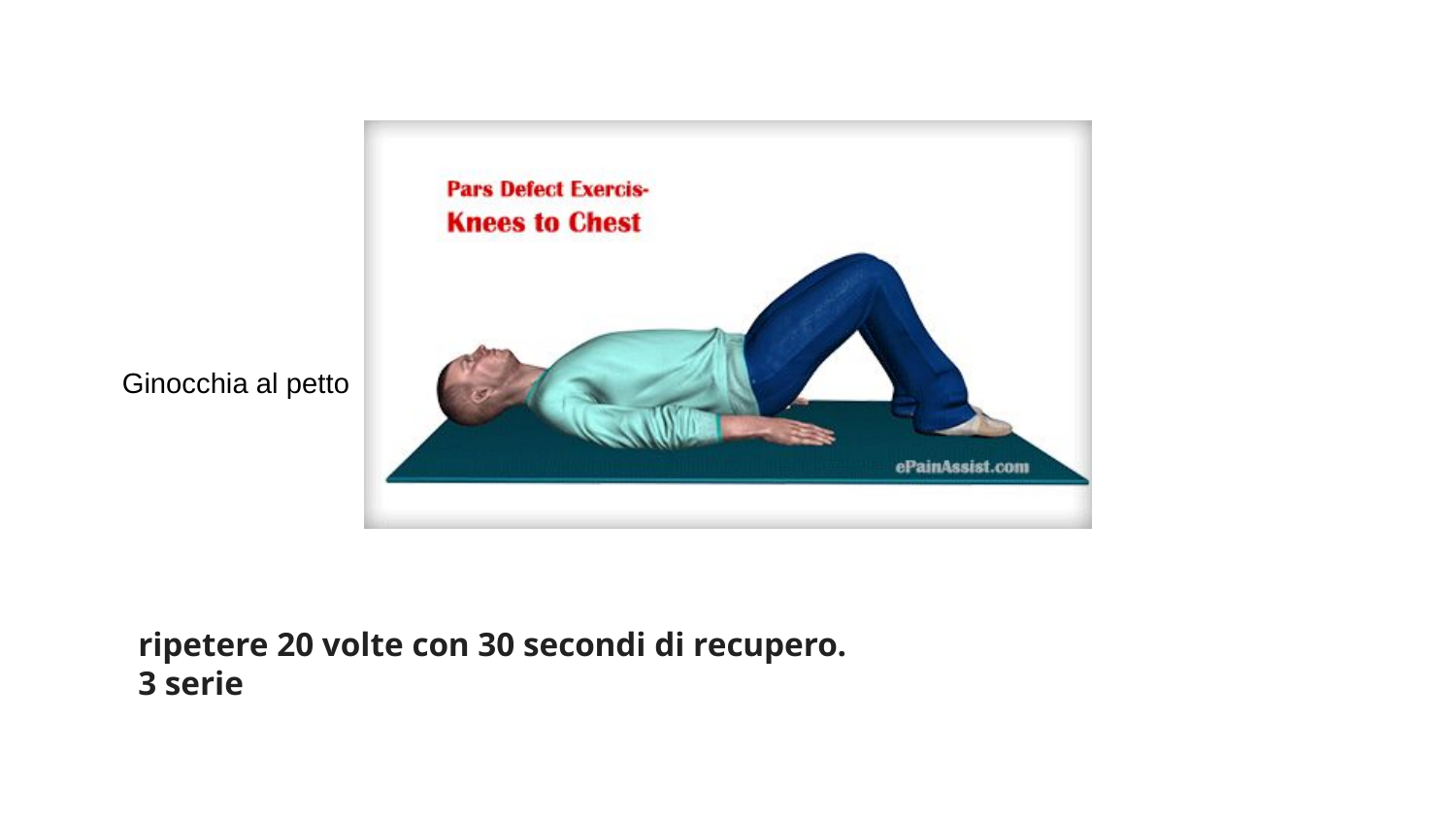

Ginocchia al petto
ripetere 20 volte con 30 secondi di recupero.
3 serie

## Slide 15
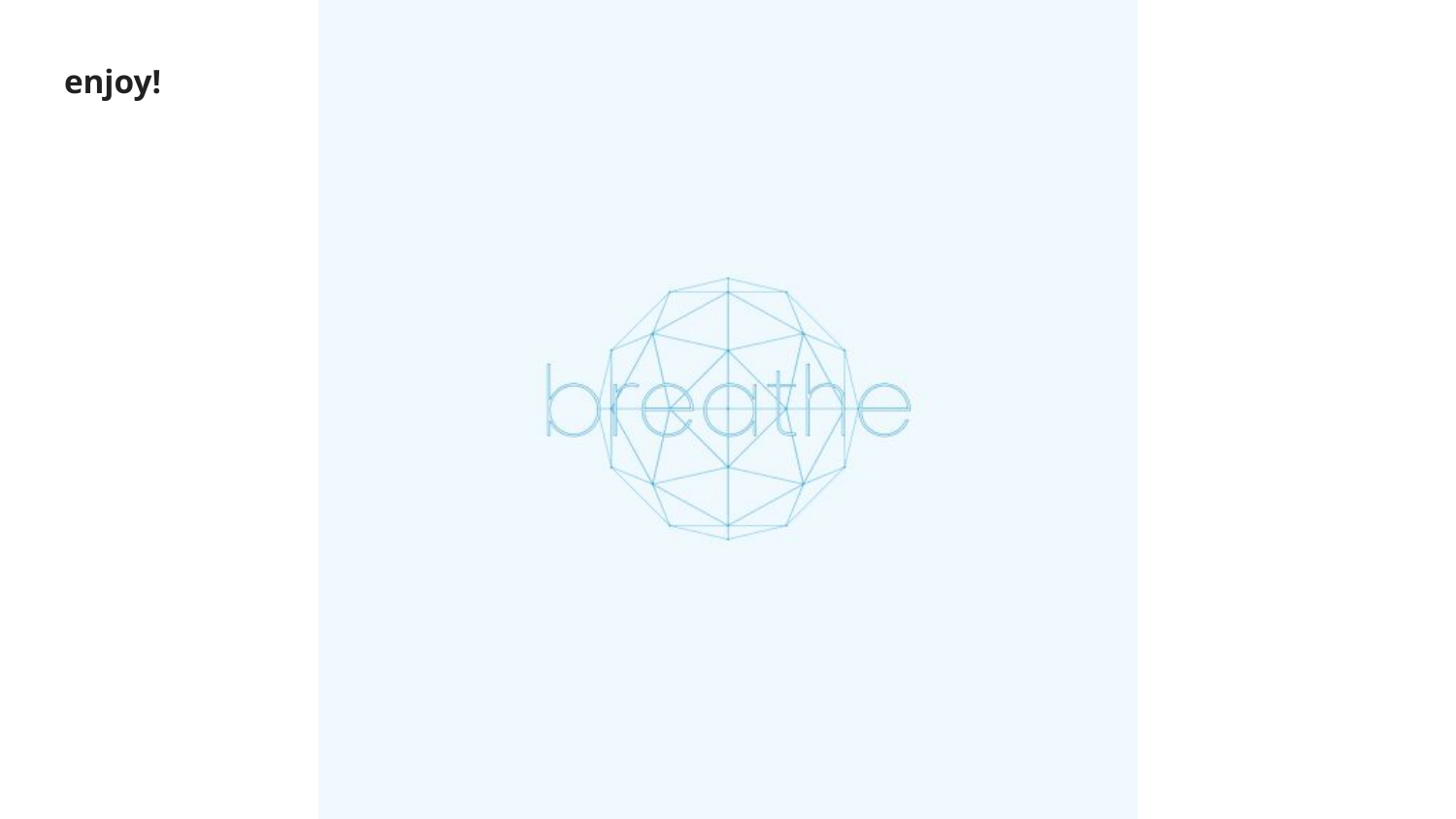

# enjoy!
